# Novel Pharmacology Following Heteromerization of the Angiotensin II Type 2 Receptor and the Bradykinin Type 2 Receptor

**DOI:** 10.3389/fendo.2022.848816

**Published:** 2022-05-26

**Authors:** Elizabeth K. M. Johnstone, Mohammed Akli Ayoub, Rebecca J. Hertzman, Heng B. See, Rekhati S. Abhayawardana, Ruth M. Seeber, Kevin D. G. Pfleger

**Affiliations:** ^1^Harry Perkins Institute of Medical Research and Centre for Medical Research, The University of Western Australia, Nedlands, WA, Australia; ^2^Australian Research Council Centre for Personalised Therapeutics Technologies, Perth, WA, Australia; ^3^School of Biomedical Sciences, The University of Western Australia, Nedlands, WA, Australia; ^4^Department of Biology, College of Science, United Arab Emirates University, Al Ain, United Arab Emirates; ^5^Dimerix Limited, Nedlands, WA, Australia

**Keywords:** angiotensin receptor, bradykinin receptor, GPCR, BRET, receptor-HIT, heteromer, NanoBRET

## Abstract

The angiotensin type 2 (AT_2_) receptor and the bradykinin type 2 (B_2_) receptor are G protein-coupled receptors (GPCRs) that have major roles in the cardiovascular system. The two receptors are known to functionally interact at various levels, and there is some evidence that the observed crosstalk may occur as a result of heteromerization. We investigated evidence for heteromerization of the AT_2_ receptor and the B_2_ receptor in HEK293FT cells using various bioluminescence resonance energy transfer (BRET)-proximity based assays, including the Receptor Heteromer Investigation Technology (Receptor-HIT) and the NanoBRET ligand-binding assay. The Receptor-HIT assay showed that Gα_q_, GRK2 and β-arrestin2 recruitment proximal to AT_2_ receptors only occurred upon B_2_ receptor coexpression and activation, all of which is indicative of AT_2_-B_2_ receptor heteromerization. Additionally, we also observed specific coupling of the B_2_ receptor with the Gα_z_ protein, and this was found only in cells coexpressing both receptors and stimulated with bradykinin. The recruitment of Gα_z_, Gα_q_, GRK2 and β-arrestin2 was inhibited by B_2_ receptor but not AT_2_ receptor antagonism, indicating the importance of B_2_ receptor activation within AT_2_-B_2_ heteromers. The close proximity between the AT_2_ receptor and B_2_ receptor at the cell surface was also demonstrated with the NanoBRET ligand-binding assay. Together, our data demonstrate functional interaction between the AT_2_ receptor and B_2_ receptor in HEK293FT cells, resulting in novel pharmacology for both receptors with regard to Gα_q_/GRK2/β-arrestin2 recruitment (AT_2_ receptor) and Gα_z_ protein coupling (B_2_ receptor). Our study has revealed a new mechanism for the enigmatic and poorly characterized AT_2_ receptor to be functionally active within cells, further illustrating the role of heteromerization in the diversity of GPCR pharmacology and signaling.

## Introduction

Angiotensin II (AngII) and bradykinin (BK) are two peptide hormones that have major regulatory roles in the cardiovascular system. AngII exerts its effects through two G protein-coupled receptors (GPCRs), the AngII type 1 (AT_1_) and the AngII type 2 (AT_2_) receptors, while BK exerts most of its cardiovascular effects through the BK type 2 (B_2_) GPCR. While the AT_1_ receptor mediates most of the classical actions of AngII, such as vasoconstriction, antinatriuresis, cell proliferation and hypertrophy (1), the effects of the AT_2_ receptor are less well characterized, and its molecular pharmacology and physiological functions remain to be fully elucidated (2, 3). Through the B_2_ receptor, BK mediates vasodilation that antagonizes the classical AngII vasoconstriction.

Although GPCRs are able to act as single, monomeric units, it is also believed that they can form homomeric or heteromeric complexes that may result in altered signaling. In particular, GPCR heteromers have been a major focus of research in GPCR pharmacology over the past decade. This has led to the characterization of numerous GPCR heteromers, including the AT_1_-AT_2_ heteromer (4–11), and also the controversial AT_1_-B_2_ heteromer (12–19). As yet, a functional heteromer between the AT_2_ and the B_2_ receptor has not been categorically demonstrated, however there are numerous examples of crosstalk between the two receptors. One of the least contentious aspects of AT_2_ receptor functioning is its action as a vasodilator. AT_2_ receptor-mediated vasodilation has been shown to occur *via* several signaling pathways, including the same nitric oxide (NO)/cyclic 3’-5’ guanosine monophosphate (cGMP) pathway involved in B_2_ receptor-mediated vasodilation (20). Furthermore, numerous studies have shown that BK is involved in AT_2_ receptor-mediated NO/cGMP vasodilation (21–23). Confocal fluorescence resonance energy transfer studies have shown the distance between the two receptors in PC12W cell membranes to be 50 ± 5 Å, suggesting that the observed functional interactions may be a result of heteromerization between the AT_2_ receptor and the B_2_ receptor (24).

**Figure 1:**
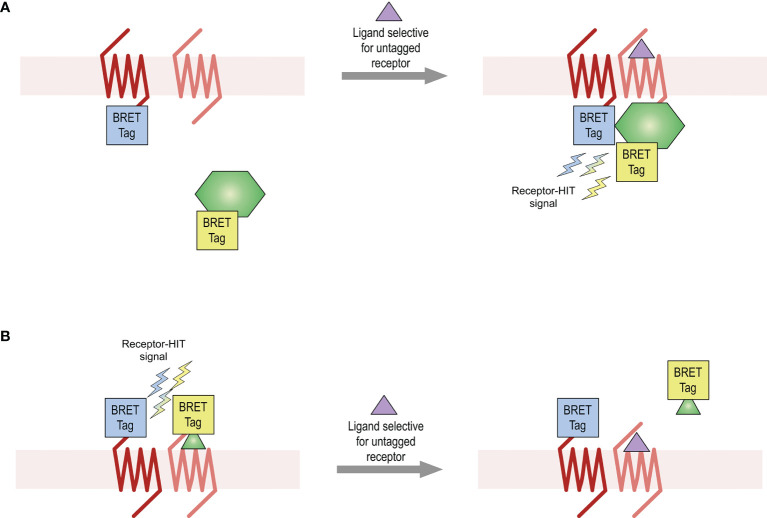
Receptor-HIT assay used for detection of receptor heteromers. The Receptor-HIT assay allows for monitoring of receptor interactions through recruitment of a labelled intracellular protein **(A)** or ligand **(B)**. In this system using BRET as the proximity assay, one receptor is fused to one BRET tag (either a luciferase or a fluorophore) while the second receptor remains untagged. The interacting biomolecule is fused with the complementary BRET tag. A BRET signal upon addition of a ligand selective for the untagged receptor is indicative of receptor heteromerization.

This study aimed to provide further evidence for the existence of the AT_2_-B_2_ heteromer in HEK293FT cells, using various bioluminescence resonance energy transfer (BRET)-based proximity assays including the Receptor-Heteromer Investigation Technology (Receptor-HIT) (25, 26) and the NanoBRET ligand binding assay (27, 28). Receptor-HIT, which has most commonly been applied to GPCRs (GPCR-HIT) (5, 25, 27, 29–32), is an assay that enables detection and characterization of heteromers through ligand-dependent interaction with biomolecules (**Figure 1**). Using various BRET assays, this study provided evidence for the existence of the AT_2_-B_2_ heteromer in our system and also revealed novel pharmacology obtained by the receptors upon heteromerization.

## Materials and Methods

### cDNA Constructs and Ligands

All receptor constructs are human unless otherwise specified. AT_2_-Rluc8 (rat) and B_2_-Rluc8 cDNA constructs were generated from plasmids containing the respective receptor cDNA tagged with Rluc. The Rluc coding region was replaced with Rluc8 cDNA from pcDNA3.1-Rluc8 kindly provided by Andreas Loening and Sanjiv Gambhir (Stanford University, CA) (33), as described previously for other constructs (34). AT_2_-Rluc (rat), AT_2_-Venus (rat) (5) and HA-AT_2_ (rat; referred to as AT_2_ in the BRET^1^ and eBRET assays) were kindly provided by Walter Thomas (University of Queensland). B_2_ and HA-B_2_ (referred to from here-on-in as B_2_) and EP_3_ cDNA was obtained from the Missouri S&T cDNA Resource Center (www.cdna.org). B_2_-Rluc was previously produced by PCR amplification of B_2_ cDNA to remove the stop codon and ligation into pcDNA_3_ containing Rluc. B_2_-Venus was generated by replacing the Rluc8 coding region from B_2_-Rluc8 with Venus cDNA. NES–Venus–mGsq was kindly provided by Nevin Lambert (Augusta University, Augusta, Georgia). Gα_q_-Rluc8, Gα_i3_-Rluc8 and Gβ_3_ were from the TRUPATH kit, which was a gift from Bryan Roth (Addgene kit #1000000163), with Venus-Gγ_9_ being generated from GFP2-Gγ_9_, also from the TRUPATH kit (35). Gα_z_-Rluc8 was kindly provided by Martina Kocan (The Florey Institute of Neuroscience and Mental Health). GRK2-Rluc8 was synthesized by GeneArt (ThermoFisher Scientific, Regensburg, Germany). The β-arrestin2-Venus cDNA construct was prepared previously from pCS2-Venus kindly provided by Atsushi Miyawaki (RIKEN Brain Science Institute, Wako-city, Japan) (34). Signal peptide and flag-tagged AT_2_ (referred to as AT_2_ in the NanoBRET ligand binding assays) and Nluc-AT_2_ were generated previously (27). Nluc-B_2_ was generated by replacing the AT_2_ coding region from Nluc-AT_2_ with B_2_ cDNA. Ligands used were AngII, BK and PGE_2_ (Sigma), icatibant and PD 123319 (Tocris Bioscience) and TAMRA-AngII (AnaSpec).

### Cell Culture and Transfection

HEK293FT cells were maintained at 37°C, 5% CO_2_ in complete medium (Dulbecco’s modified Eagle’s medium (DMEM) containing 0.3 mg/ml glutamine, 100 IU/ml penicillin, and 100 µg/ml streptomycin) supplemented with 10% fetal calf serum (FCS) (GIBCO BRL, Carlsbad, CA). Transient transfections were carried using either GeneJuice (Merck, Kilsyth, Australia) or FuGENE (Promega) according to manufacturer’s instructions. All assays were carried out 48 hours post transfection.

### Receptor-HIT

Receptor-HIT is an assay that enables the identification and pharmacological profiling of receptor heteromers in live cell systems. The assay uses a proximity-based reporter system such as BRET to enable detection of heteromers through their ligand-dependent interaction with proteins or ligands (5, 25, 27, 29–32). The Receptor-HIT assay comprises three elements (**Figure 1**), which in these studies on the BRET platform are a BRET-tagged receptor, an untagged receptor, and a BRET-tagged interacting protein (**Figure 1A**) or a BRET-tagged interacting ligand [**Figure 1B** (27)]. If a change in BRET signal occurs upon addition of a ligand that is selective for the untagged receptor, this indicates proximity between the tagged receptor and the tagged interacting biomolecule. This Receptor-HIT signal signifies the close proximity of the two receptors, and is indicative of receptor heteromerization.

### BRET^1^ and eBRET Assays

HEK293FT cells were transfected with cDNA as described in figure legends. BRET^1^ and eBRET assays used rat AT_2_ constructs. For all BRET^1^ assays (with the exception of **Figures 6E, F**), 5 µM coelenterazine *h* (Promega) was added and basal BRET was measured for 10-20 mins before adding agonist or vehicle and then continuing to measure BRET. Antagonist assays had a pretreatment of antagonist or vehicle (30 min) prior to addition of coelenterazine *h.* For the BRET^1^ assays in **Figures 6E, F**, cells were pretreated for 30 min with agonist or vehicle, with cells in **Figure 6F** having an additional pretreatment of antagonist or vehicle (30 min) prior to treatment with agonist. Following pretreatment, coelenterazine *h* was added to a final concentration of 5 µM and BRET was measured immediately. For eBRET assays, cells were incubated at 37°C, 5% CO_2_ for 2 hours with 30 µM EnduRen (Promega) to ensure substrate equilibrium was reached. Basal BRET was measured for 10-20 mins before adding agonist or vehicle and then continuing to measure BRET. Antagonist assays had a pretreatment of antagonist or vehicle (30 min) prior to addition of coelenterazine *h.* All BRET^1^ and eBRET measurements were taken at 37°C using either a LUMIstar Omega plate reader (BMG Labtech, Mornington, Victoria, Australia) with 460–490 nm and 520–550 nm filters; a CLARIOstar plate reader (BMG Labtech) with 420–480 nm and 520–620 nm filters; or a VICTOR Light plate reader (Perkin Elmer) with 400–475 nm and 520–540 nm filters. The ligand-induced BRET signal was calculated by subtracting the ratio of the long wavelength emission over the short wavelength emission for a vehicle-treated cell sample from the same ratio for a second aliquot of the same cells treated with agonist, as described previously (34, 36). In this calculation, the vehicle-treated cell sample represents the background, eliminating the requirement for measuring a donor-only control sample (34, 36). For BRET kinetic assays, the final pretreatment reading is presented at the zero time point (time of agonist/vehicle addition).

### NanoBRET Assays

HEK293FT cells were transfected with cDNA as described in figure legends. NanoBRET assays used human AT_2_ constructs. For NanoBRET assays, cells were pretreated for 30 min with PD 123319 and then TAMRA-AngII was added (final concentration of 1 μM). Following another 30 min incubation, furimazine was added and BRET measured immediately at 37°C using a PHERAstar *FS* plate reader (BMG Labtech) with 420–500 nm and 610–LP filters or a LUMIstar Omega plate reader (BMG Labtech) with 410–490 nm and 610–LP filters. The BRET signal was calculated by subtracting the ratio of the long wavelength emission over the short wavelength emission and the data were normalized as percentage of TAMRA-AngII binding.

### IP_1_ Accumulation Assays

Measurement of IP_1_ accumulation was performed using the IP-One Tb kit (Cisbio Bioassays) according to manufacturer’s instructions. Cells were treated for 30 minutes at 37°C with agonists or vehicle. Antagonist assays had an additional pre-treatment with antagonist or vehicle for 30 mins at 37°C, which was removed prior to treatment with agonist. The cells were then lysed by adding the supplied assay reagents, and the assay was incubated for 1 hour at room temperature. Fluorescence was measured at 620 nm and 665 nm 50 µs after excitation at 340 nm using the EnVision 2102 multilabel plate reader (PerkinElmer).

### Data Presentation and Statistical Analysis

Data were presented and analyzed using Prism 9 software (GraphPad). Competition binding data and concentration-response data were fitted using logarithmic nonlinear regression (three parameter). Unpaired *t*-tests, one-way ANOVAs and two-way ANOVAs were used to determine statistical significance where appropriate (*p < 0.05).

## Results

### Gα_q_ Coupling to the AT_2_-B_2_ Heteromer

**Figure 2:**
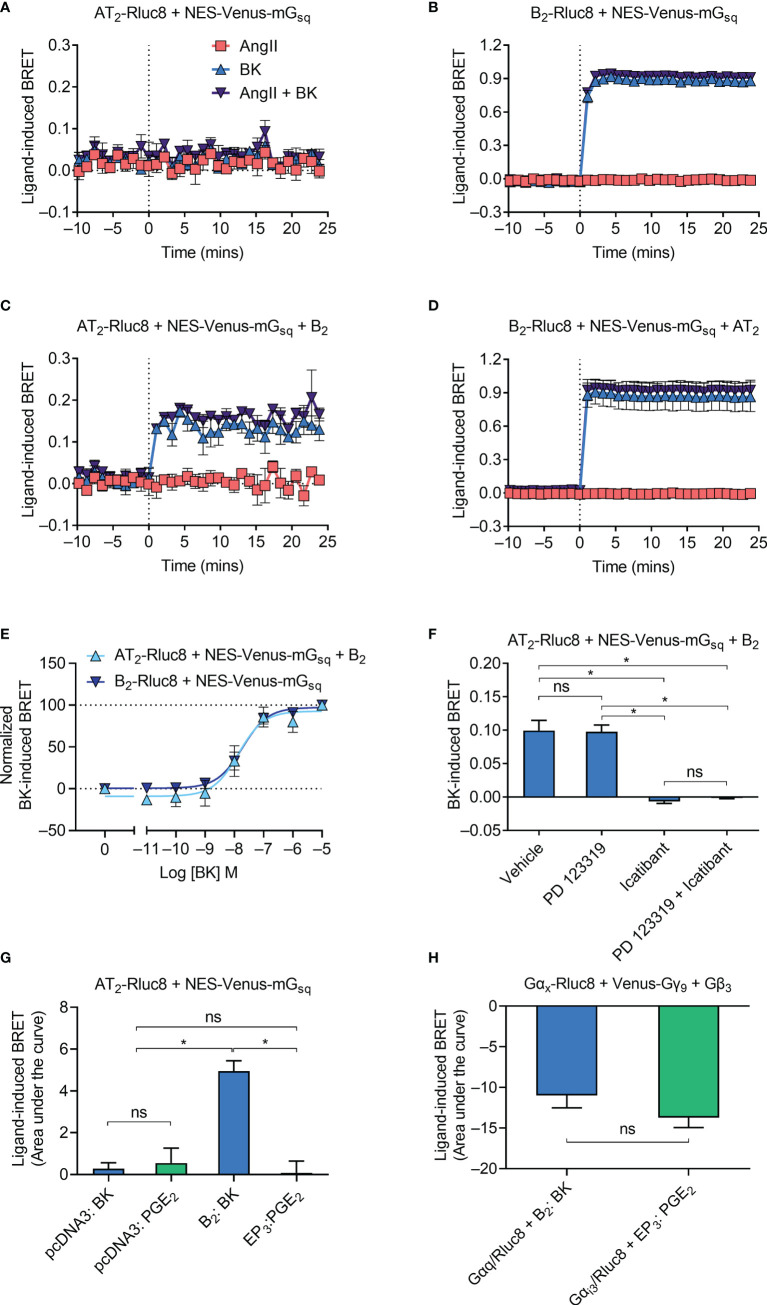
Gα_q_ recruitment to the AT_2_-B_2_ heteromer. HEK293FT cells were transfected with plasmid cDNA as described on graphs. **(A–D)** Time course analysis showing recruitment of NES-Venus-mG_sq_ to receptors following addition of ligands at 0 mins. **(E)** BK concentration-response analysis showing recruitment of NES-Venus-mG_sq_ to receptors. Normalized data taken from BRET assays at 17 min after agonist addition. **(F)** NES-Venus-mG_sq_ Receptor-HIT assay in the presence of 50 μM antagonists (or vehicle) and 0.1 μM BK. Data are from BRET assays at 17 min after agonist addition. *p < 0.05; ns, not significant (one-way ANOVA with Tukey’s multiple comparisons test). **(G)** Area under the curve ligand-induced BRET data. *p < 0.05; ns, not significant (one-way ANOVA with Tukey’s multiple comparisons test). **(H)** Area under the curve ligand-induced BRET data. *p < 0.05; ns, not significant (unpaired *t*-test). All data are presented as mean ± SEM of ≥ three independent experiments performed in triplicate.

Following activation by an agonist, GPCRs typically interact with and activate heterotrimeric G proteins to initiate intracellular signaling cascades. The B_2_ receptor primarily couples to the Gα_q_ class of G proteins (37) while the AT_2_ receptor is an unusual GPCR in that it does not readily couple to any G proteins (38). To investigate Gα_q_ coupling by the receptors, we used a Venus-tagged mini G (mG) protein construct that comprises an engineered GTPase domain of the Gα_s_ protein that has been modified to confer Gα_q_ specificity (NES-Venus-mG_sq_). As expected, no ligand-induced recruitment of NES-Venus-mG_sq_ to the Rluc8-tagged AT_2_ receptor (AT_2_-Rluc8) was observed (**Figure 2A**). In contrast, and also as expected, coexpression of NES-Venus-mG_sq_ with the B_2_ receptor tagged with Rluc8 (B_2_-Rluc8) resulted in a BK-induced BRET signal (**Figure 2B**) indicative of recruitment of Gα_q_ to the receptor.

We then investigated Gα_q_ coupling using the Receptor-HIT assay, again using NES-Venus-mG_sq_. Receptor-HIT uses a proximity-based reporter system such as BRET to enable detection and characterization of heteromers through their ligand-dependent interactions with labelled proteins or ligands (5, 25, 29–32) (**Figure 1**). Upon coexpression of the unlabeled B_2_ receptor in cells expressing AT_2_-Rluc8 and NES-Venus-mG_sq_ we now observed a BK-induced BRET signal (**Figure 2C**), indicating recruitment of NES-Venus-mG_sq_ proximal to AT_2_-Rluc8. This Receptor-HIT signal indicates the close proximity of the AT_2_ receptor and the B_2_ receptor, and suggests their interaction within a heteromeric complex. Coexpression of untagged AT_2_ receptor to cells expressing B_2_-Rluc8 and NES-Venus-mG_sq_ did not alter the BRET signal (**Figure 2D**) from that seen without AT_2_ expression (**Figure 2B**).

We investigated the mG_sq_ Receptor-HIT signal further by conducting concentration-response analysis. **Figure 2E** shows that there is no change in potency of mG_sq_ coupling to AT_2_-B_2_ heteromers compared to B_2_ receptors (pEC_50_ ± SEM = 7.94 ± 0.29 vs. 7.73 ± 0.19, respectively; p > 0.05, unpaired *t*-test). When we conducted the mG_sq_ Receptor-HIT assay in the presence of selective antagonists (**Figure 2F**), we saw that the AT_2_ receptor antagonist PD 123319 did not inhibit coupling of mG_sq_ to AT_2_-B_2_ heteromers. In contrast, the putative B_2_ receptor antagonist icatibant was able to significantly reduce the level of mG_sq_ recruitment, indicating the requirement of B_2_ receptor activation for Gα_q_ coupling.

Finally, we investigated the specificity of the Receptor-HIT signal by conducting a similar experiment but instead using a GPCR not known to heteromerize with the AT_2_ receptor, the prostaglandin E receptor 3 (EP_3_ receptor). Here we found that only coexpression and activation of the B_2_ receptor resulted in a Receptor-HIT signal between AT_2_-Rluc8 and NES-Venus-mG_sq_ (**Figure 2G**). No signal was observed when EP_3_ was coexpressed with AT_2_-Rluc8 and NES-Venus-mG_sq_ and treated with PGE_2_ (**Figure 2G**), despite both B_2_ and EP_3_ being expressed within the cells, as shown by their activation of G protein (**Figure 2H**; Gα_q_ for B_2_, and Gα_i3_ for EP_3_).

### Activation of the IP_1_ Signaling Pathway

**Figure 3:**
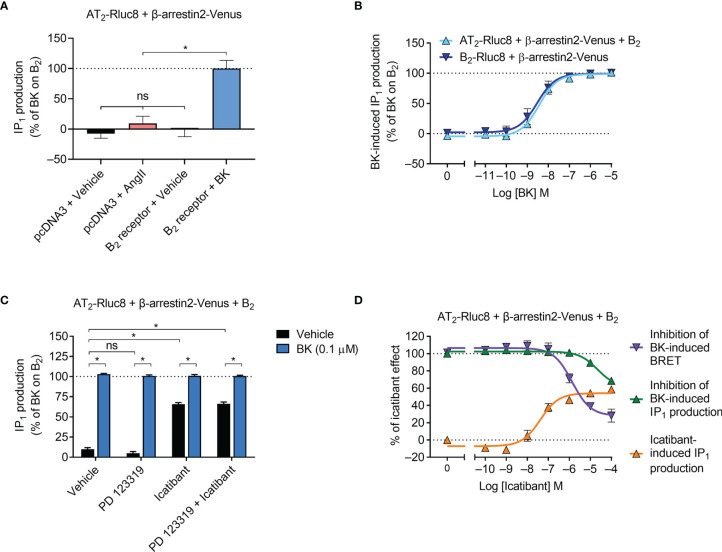
IP_1_ signaling by the AT_2_-B_2_ heteromer. HEK293FT cells were transfected with plasmid cDNA as described on graphs. **(A)** Ligand-induced IP_1_ signaling in cells expressing AT_2_-Rluc8, with or without the B_2_ receptor. *p < 0.05; ns, not significant (one-way ANOVA with Tukey’s multiple comparisons test). **(B)** Concentration-response analysis showing BK-induced IP_1_ signaling. **(C)** IP_1_ assay in the presence of 10 μM antagonists (or vehicle) and 0.1 μM BK. *p < 0.05 (two-way ANOVA with Sidak’s multiple comparisons test). **(D)** Concentration-response analysis comparing agonistic and antagonistic actions of icatibant, the latter antagonizing 0.1 μM BK. All data are presented as mean ± SEM of ≥ three independent experiments performed in duplicate (IP_1_ assay) or triplicate (BRET).

Gα_q_ activation initiates a signaling cascade that leads to inositol phosphate signaling, which can be monitored by measuring the accumulation of the metabolite IP_1_. Using an IP_1_ assay and aliquots of transfected cells also used in the β-arrestin2 assays described below, we next investigated downstream Gα_q_ signaling mediated by the receptors. As expected, we found that AngII did not induce IP_1_ production in cells expressing AT_2_/Rluc8 (**Figure 3A**). However, coexpression of the B_2_ receptor resulted in robust BK-induced IP_1_ production (**Figure 3A**). When we conducted concentration-response analysis, we found that there was no significant difference in the potency of IP_1_ production between cells expressing just the B_2_ receptor and cells expressing the B_2_ receptor and the AT_2_ receptor (**Figure 3B**; pEC_50_ ± SEM = 8.55 ± 0.20 vs. 8.45 ± 0.04, respectively; p > 0.05, unpaired *t*-test), just as we saw no difference in potency of mG_sq_ recruitment in the BRET assay. When we conducted these IP_1_ assays with an antagonist pretreatment, we found that 10 μM of the AT_2_ receptor antagonist PD 123319 had no inhibitory effect on 0.1 μM BK-induced IP_1_ production (**Figure 3C**). Interestingly, in this assay 10 μM of the putative B_2_ selective antagonist icatibant was also unable to inhibit 0.1 μM BK-induced IP_1_ production. Indeed, it acted as a partial agonist in this assay, as can be seen by the substantial IP_1_ production in cells treated only with icatibant and no BK. Further analysis illustrated the concentration-dependent effect of IP_1_ production mediated by icatibant (**Figure 3D**). This concentration-response analysis also showed that high concentrations of icatibant were in fact able to inhibit BK-induced IP_1_ production. However, the potency of this effect was shifted substantially to the right of its inhibitory actions on BK-induced β-arrestin2 recruitment. These findings support reports of the partial agonism of icatibant, which has previously been observed mediating IP_1_ production through the B_2_ receptor (39).

### Gα_z_ Recruitment to the AT_2_-B_2_ Heteromer

**Figure 4:**
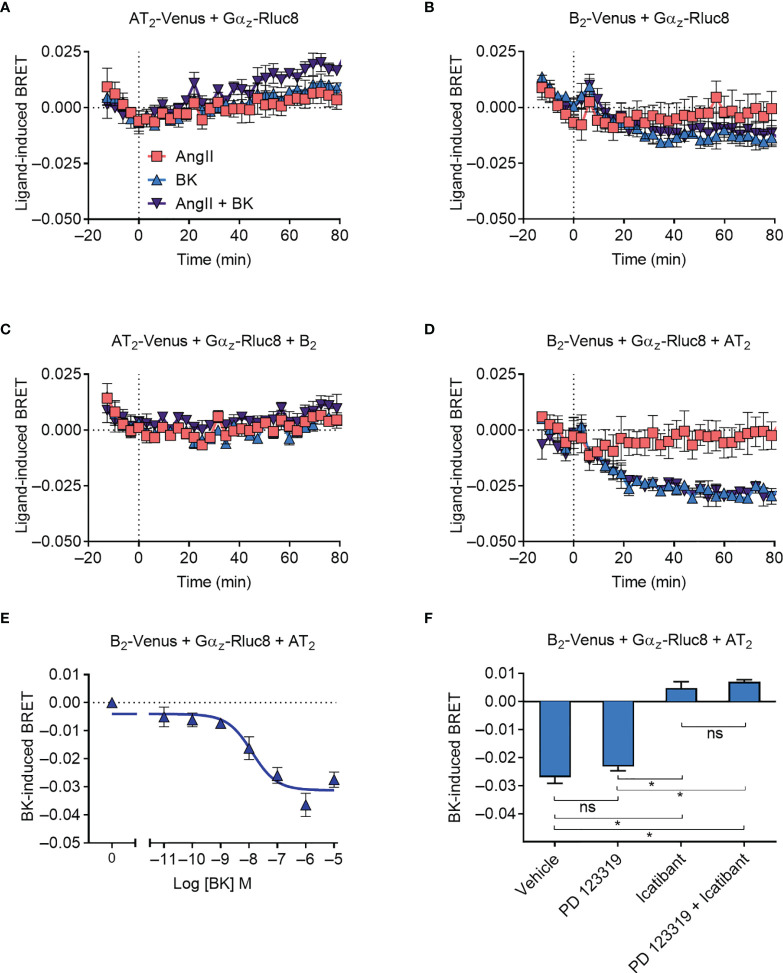
Gα_z_ recruitment to the AT_2_-B_2_ heteromer. HEK293FT cells were transfected with plasmid cDNA as described on graphs. **(A–D)** Time course analysis showing interaction of Gα_z_-Rluc8 with receptors following addition of ligands at 0 mins. **(E)** BK concentration-response analysis showing recruitment of Gα_z_-Rluc8 to B_2_ receptors. Data taken from BRET assays at 60 min after agonist addition. **(F)** Gα_z_-Rluc8 Receptor-HIT assay in the presence of 10 μM antagonists and 0.1 μM BK. Data taken from BRET assays at 30 min after agonist addition. *p < 0.05; ns, not significant (one-way ANOVA with Tukey’s multiple comparisons test). All data are presented as mean ± SEM of ≥ three independent experiments performed in triplicate.

We next investigated Gα_z_ protein recruitment to the receptors, using Gα_z_ tagged with Rluc8 (Gα_z_-Rluc8). As Gα_z_ is not a known signaling partner for either the AT_2_ receptor or the B_2_ receptor, we did not expect to observe any recruitment, and this was confirmed in our BRET assay expressing either Venus-tagged receptor and Gα_z_-Rluc8 (**Figures 4A, B**). Coexpression of the untagged B_2_ receptor did not alter the BRET signal between AT_2_-Venus and Gα_z_-Rluc8 (**Figure 4C**), however, coexpression of the untagged AT_2_ receptor interestingly resulted in a marked decrease in the BRET signal between B_2_-Venus and Gα_z_-Rluc8 upon treatment with BK (**Figure 4D**). A decrease in the BRET signal suggests that there is a preformed complex between B_2_-Venus and Gα_z_-Rluc8, which either disassociates or undergoes conformational rearrangement that increases the distance between the two BRET tags (40–42). In either case, this BRET signal provides further evidence in support of the existence of a functional AT_2_-B_2_ heteromer, and illustrates completely novel pharmacology it has adopted.

We also investigated the concentration-dependence of the Gα_z_ BRET signal (**Figure 4E**) and found a similar potency of BK-induced concentration-dependence as observed for mG_sq_ coupling (pEC_50_ ± SEM = 7.94 ± 0.29, unpaired *t*-test). Likewise, when we conducted the assay in the presence of selective antagonists, we again found that the Gα_z_ BRET signal could be blocked by B_2_ receptor inhibition (icatibant), but not AT_2_ inhibition (PD 123319) (**Figure 4F**).

### GPCR Kinase 2 Recruitment to the AT_2_-B_2_ Heteromer

**Figure 5:**
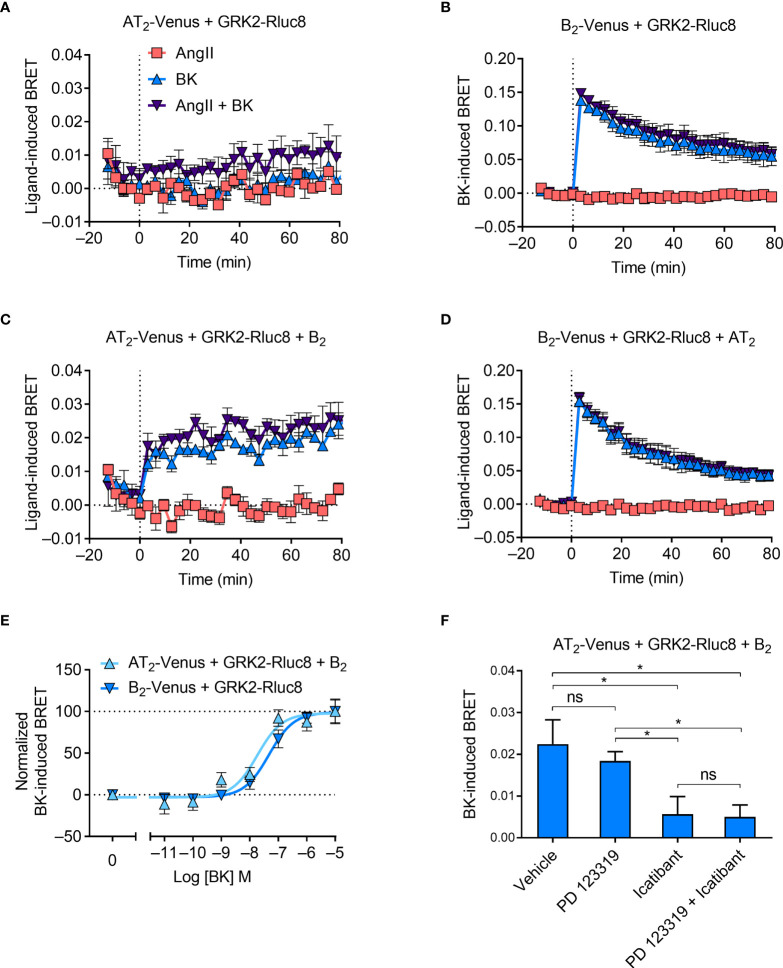
GRK2 recruitment to the AT_2_-B_2_ heteromer. HEK293FT cells were transfected with plasmid cDNA as described on graphs. **(A–D)** Time course analysis showing recruitment of GRK2-Rluc8 to receptors following addition of ligands at 0 mins. **(E)** Concentration-response analysis showing recruitment of GRK2-Rluc8 to receptors. Normalized data taken from BRET assays at 10 min after agonist addition. **(F)** GRK2-Rluc8 Receptor-HIT assay in the presence of 50 μM antagonists and 1 μM BK. Data are from BRET assays at 20 min after agonist addition. *p < 0.05; ns, not significant (one-way ANOVA with Tukey’s multiple comparisons test). All data are presented as mean ± SEM of ≥ three independent experiments performed in triplicate.

Following agonist stimulation, GPCR kinases (GRKs) are rapidly recruited to GPCRs, where they phosphorylate the receptor’s C terminal tail. This initiates receptor desensitization and interaction with β-arrestin proteins. We investigated GRK recruitment using BRET with Rluc8-tagged GRK2 (GRK2-Rluc8) and Venus-tagged receptors. There was no ligand-induced recruitment of GRK2-Rluc8 to the Venus-tagged AT_2_ receptor (AT_2_-Venus; **Figure 5A**). This lack of GRK2 recruitment is expected, as it is well known that the AT_2_ receptor does not recruit β-arrestin or internalize upon stimulation with AngII (43–45), and therefore it is unlikely it would recruit GRKs. In contrast, but also as expected, when cells expressing GRK2-Rluc8 and Venus-tagged B_2_ receptor (B_2_-Venus) were treated with BK (but not AngII) we saw an immediate increase in the BRET signal, indicating rapid recruitment of GRK2 to the B_2_ receptor (**Figure 5B**).

When we coexpressed untagged B_2_ receptor in cells expressing AT_2_-Venus and GRK2-Rluc8, we saw BK-induced recruitment of GRK2 proximal to the AT_2_ receptor (**Figure 5C**). Interestingly, this BRET signal had a much more sustained signal than that observed between GRK2-Rluc8 and B_2_-Venus (**Figure 5B**), which declined steadily over time. As with the mG_sq_ Receptor-HIT assay, this Receptor-HIT signal indicates the close proximity of the AT_2_ receptor and the B_2_ receptor, and suggests their interaction within a heteromeric complex. Coexpression of untagged AT_2_ receptor did not alter the BRET signal between B_2_-Venus and GRK2-Rluc8 (**Figure 5D**) from that seen without AT_2_ expression (**Figure 5B**).

We also investigated the GRK2 Receptor-HIT signal further by conducting concentration-response analysis. **Figure 5E** shows that there is a significant leftward shift in the potency of GRK2 recruitment to AT_2_-B_2_ heteromers compared to B_2_ receptors (pEC_50_ ± SEM = 8.07 ± 008 vs. 7.35 ± 0.06, respectively; p < 0.05, unpaired *t*-test). When we conducted the GRK2 Receptor-HIT assay in the presence of selective antagonists (**Figure 5F**) we saw, as in the mG_sq_ and Gα_z_ Receptor-HIT assays, that the AT_2_ receptor antagonism did not inhibit the recruitment of GRK2 to AT_2_-B_2_ heteromers, while B_2_ receptor antagonism significantly reduced the level of GRK2 recruitment, indicating the specificity of the BRET signals.

### β-arrestin2 Recruitment to the AT_2_-B_2_ Heteromer

Following GRK recruitment and subsequent receptor phosphorylation, GPCRs recruit the scaffold protein β-arrestin, which desensitizes the receptor from classical cell surface G protein signaling and initiates internalization (46). Individual GPCRs have different β-arrestin recruitment profiles resulting in unique desensitization and internalization characteristics. Upon treatment with BK, the B_2_ receptor rapidly recruits β-arrestin leading to swift desensitization and extensive internalization (47, 48). In contrast and as already mentioned, the AT_2_ receptor does not recruit β-arrestin or internalize upon stimulation with AngII (43–45).

**Figure 6:**
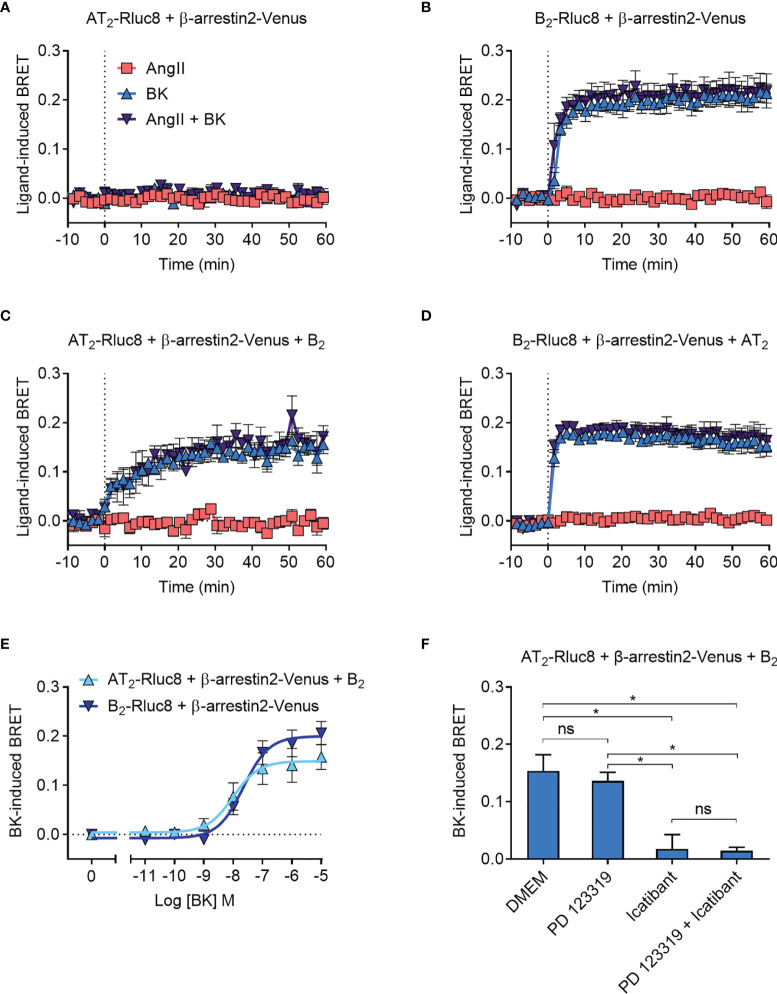
β-arrestin2-Venus recruitment to the AT_2_-B_2_ heteromer. HEK293FT cells were transfected with plasmid cDNA as described on graphs. **(A–D)** Time course analysis showing recruitment of β-arrestin2-Venus to receptors following addition of ligands at 0 mins. **(E)** Concentration-response analysis showing recruitment of β-arrestin2-Venus to receptors. Data taken from BRET assays at 40 min after agonist addition. **(F)** β-arrestin2-Venus Receptor-HIT assay in the presence of 10 μM antagonists and 0.1 μM BK. Data taken from BRET assays at 40 min after agonist addition. *p < 0.05; ns, not significant (one-way ANOVA with Tukey’s multiple comparisons test). All data are presented as mean ± SEM of ≥ three independent experiments performed in triplicate.

As expected, there was no ligand-induced recruitment of β-arrestin2-Venus to AT_2_-Rluc8 (**Figure 6A**), whereas when we coexpressed B_2_-Rluc8 with β-arrestin2 tagged with Venus (β-arrestin2-Venus) we observed strong and rapid BK-induced recruitment of β-arrestin2-Venus to B_2_-Rluc8 (**Figure 6B**). When AT_2_-Rluc8 was co-expressed with the untagged B_2_ receptor in the Receptor-HIT configuration, there was a marked increase in ligand-induced BRET when the cells were treated with BK but not AngII (**Figure 6C**), indicating BK-dependent translocation of β-arrestin2-Venus proximal to the B_2_ receptor. This BRET signal between AT_2_-Rluc8 and β-arrestin2-Venus confirms the close proximity of AT_2_-Rluc8 and the B_2_/β-arrestin2-Venus complex and is indicative of AT_2_-B_2_ heteromerization. Additionally, and similar to what was seen with GRK2, β-arrestin2-Venus recruitment to AT_2_-B_2_ heteromers had an altered kinetic profile to what was seen with B_2_ monomers/homomers. When we conducted the Receptor-HIT assay in the reverse configuration by coexpressing the untagged AT_2_ receptor with B_2_-Rluc8 and β-arrestin2-Venus there was no change in BRET signal (**Figure 6D**) from that seen without AT_2_ expression (**Figure 6B**).

To further investigate the β-arrestin2 Receptor-HIT signal we again conducted concentration-response analysis (**Figure 6E**). This showed that there was no significant difference in potency between BK-induced β-arrestin2-Venus recruitment to B_2_ receptors and AT_2_-B_2_ heteromers (pEC_50_ ± SEM = 7.64 ± 0.06 vs. 7.94 ± 0.19, respectively; p > 0.05, unpaired *t*-test). We then conducted the β-arrestin2 Receptor-HIT assay in the presence of selective antagonists. Similar to the previous Receptor-HIT assays, we saw that the BK-induced recruitment of β-arrestin2 to the AT_2_-B_2_ heteromer could be blocked by B_2_ receptor inhibition but not AT_2_ inhibition (**Figure 6F**), which demonstrates the importance of B_2_ receptor coexpression and activation.

### NanoBRET Ligand Binding to the AT_2_-B_2_ Heteromer

**Figure 7:**
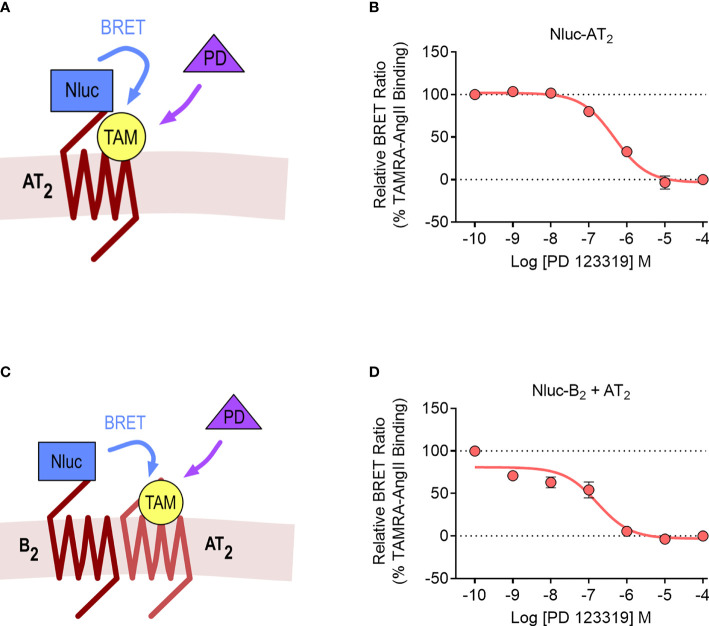
NanoBRET assay for detection of ligand binding to the AT_2_ receptor and the AT_2_-B_2_ heteromer. Depiction of the NanoBRET assay for detection of TAMRA-AngII (TAM) ligand binding to Nluc-AT_2_ **(A)** and AT_2_ receptors heteromerized with B_2_ receptors (using the Receptor-HIT assay) **(C)**. HEK293FT cells were transfected with Nluc-AT_2_ and pcDNA3 **(B)** or Nluc-B_2_ and AT_2_ **(D)** and competition binding assays were conducted with TAMRA-AngII and PD 123319. Data are presented as mean ± SEM of ≥ three independent experiments performed in duplicate.

We lastly investigated the AT_2_-B_2_ heteromer using the NanoBRET ligand binding assay (28). In this assay, the NanoLuc (Nluc) luciferase (49) is fused to the N-terminus of a GPCR, and binding of fluorescent ligands can be detected with BRET. In our study, we fused Nluc to the N-terminus of the AT_2_ receptor (Nluc-AT_2_) and treated cells with an AngII analogue tagged with the TAMRA fluorophore (TAMRA-AngII) (**Figures 7A, B**). When we treated cells with increasing concentrations of the AT_2_ receptor antagonist PD 123319 in a competition binding assay, we were able to see a reduction in the BRET signal, indicating displacement of TAMRA-AngII binding to Nluc-AT_2_.

We then conducted the ligand binding assay in the Receptor-HIT configuration, as recently published (27). Here, Nluc was fused to the N-terminus of the B_2_ receptor (Nluc-B_2_) and was coexpressed with the untagged AT_2_ receptor. A BRET signal upon addition of TAMRA-AngII would indicate both the binding of TAMRA-AngII to the untagged AT_2_ receptor and also its close proximity to Nluc-B_2_, and this specific binding of TAMRA-AngII to AT_2_ receptors would be confirmed by displacement with PD 123319 (**Figure 7C**). When we conducted the assay, this was precisely what we observed, a TAMRA-AngII-induced BRET signal that could be displaced by increasing concentrations of PD 123319 (**Figure 7D**). When we compared the pIC_50_ values between cells expressing Nluc-AT_2_ and cells expressing Nluc-B_2_ and AT_2_ we found no significant differences (pIC_50_ ± SEM = 6.33 ± 0.10 vs. 6.84 ± 0.20, respectively; p > 0.05, unpaired *t*-test).

## Discussion

This study provides evidence for the existence of the AT_2_-B_2_ receptor heteromer in transfected HEK293FT cells. This is illustrated by the Receptor-HIT signals that show the requirement of B_2_ receptor coexpression and activation for recruitment of mG_sq_, GRK2 and β-arrestin2 proximal to the AT_2_ receptor. Evidence also came from the Gα_z_ assay that demonstrated BK-induced modulation of B_2_ receptor/Gα_z_ coupling, which was not present without AT_2_ receptor coexpression. Finally, the results of the heteromer ligand binding assay confirmed the close proximity of the two receptors at the cell surface, showing a Receptor-HIT signal between Nluc-B_2_ and TAMRA-AngII bound to AT_2_ receptors.

Perhaps the most interesting finding of this study was the novel G protein signaling pharmacology observed in the form of BK-induced modulation of B_2_ receptor/Gα_z_ coupling that was not present without AT_2_ receptor coexpression. Following a search of the literature, we were unable to find any evidence that either the AT_2_ or the B_2_ receptor individually couple to Gα_z_, and this fits with the lack of ligand-induced interaction we observed in our BRET assays expressing only the single receptor. It is therefore particularly interesting that heteromerization may lead to new G protein coupling for both receptors. The Gα_z_ protein is in the Gα_i/o_ class of G proteins and therefore its canonical effect is inhibition of adenylyl cyclase and cAMP signaling (50). Gene and protein expression studies show that it is expressed at particularly high levels in the nervous system, and also at detectable levels in the gastrointestinal and reproductive systems as well as the adrenal gland and smooth muscle tissue (50, 51). There is therefore some overlap in expression profiles with the AT_2_ receptor and the B_2_ receptor, both of which are expressed in the brain, vasculature, adrenal gland and reproductive tissues (37, 38, 52). This suggests that the AT_2_-B_2_ heteromer could have physiological roles outside of the cardiovascular system, which is where most of the research into functional interactions between the two receptors has primarily been focused. In particular, the coexpression of Gα_z_ and the two receptors in the nervous system is especially interesting, due to the growing appreciation of the functional role of the AT_2_ receptor in mediating neurological processes (53, 54). Indeed, an AT_2_ receptor antagonist progressed to Phase II clinical trials for the treatment of neuropathic pain (54, 55), although the trial had to be terminated due to toxicological concerns arising from pre-clinical data that only became available after the start of the trial (56). In addition, it is well established that the B_2_ receptor is also involved in the mediation of various types of pain, including neuropathic pain (57), and it is interesting to speculate on a possible involvement of the AT_2_-B_2_ heteromer in mediating pain or other neurological processes.

The AT_2_ receptor is an unusual GPCR in that it does not readily signal through G proteins, and nor does it undergo agonist-induced desensitization or internalization. In addition, despite decades of research, even the physiological effects mediated by the AT_2_ receptor are still not well understood. It is most commonly believed to antagonize many of the characteristic AngII/AT_1_-mediated actions, such as vasoconstriction, anti-natriuresis, growth and cell proliferation. However, numerous studies describe opposing effects of the AT_2_ receptor, reporting its mediation, rather than opposition of these effects (3). Despite these conflicting studies, one of the least controversial aspects of AT_2_ receptor pharmacology is its action as a vasodilator, *via* stimulation of the NO/cGMP signaling pathway (58). This is the same pathway used by the B_2_ receptor to mediate vasodilation following BK-induced activation of Gα_q_ signaling (59). It is well known that BK can be involved in AT_2_ receptor-mediated NO signaling (22, 23), and the results of our study may provide further insight into this signaling cascade, demonstrating that Gα_q_ can be recruited proximal to the AT_2_ receptor when it is heteromerized with the B_2_ receptor. Furthermore, a previous study has suggested that functional heteromerisation of these two receptors leads to enhanced NO signaling (24). The recruitment of mG_sq_ proximal to the AT_2_ receptor within the AT_2_-B_2_ heteromer in our study, potentially provides a mechanism for this enhanced NO signal, as BK-mediated NO signaling could be mediated not only by B_2_ receptors but also by AT_2_-B_2_ heteromers. It is important to note that not all AT_2_ receptor-mediated NO signaling requires the presence of B_2_ receptors, as B_2_ receptor knockout mice can produce NO directly from AT_2_ receptors (21). This therefore indicates an alternate pathway used by the AT_2_ receptor to mediate NO signaling, which does not require the presence of B_2_ receptors, and may therefore not necessarily be directly impacted by AT_2_-B_2_ heteromerization.

An interesting recent study has revealed that β-arrestin2 is an integral component of an endothelial NO synthase (eNOS) signaling pathway (60). Here, β-arrestin2 was found to colocalize in sinusoidal endothelial cells with GPCR kinase interactor 1 and eNOS, stimulating eNOS activity in a ERK1/2- and Src-dependent manner. The study revealed that endothelin-1-mediated eNOS activity required β-arrestin2, and therefore it is likely that it is also involved in NO signaling by both the AT_2_ and the B_2_ receptor, as well as the AT_2_-B_2_ heteromer.

It is now well established that endocytosed GPCR-bound β-arrestins are able to aid in the initiation of signaling cascades through their function as scaffold proteins. Numerous signaling molecules are regulated through this property of β-arrestins, such as the MAPKs ERK, JNK, and p38. The AT_2_ receptor most commonly exerts inhibitory effects on MAPK cascades through activation of phosphatases (61–64). The potential for additional signaling through β-arrestin scaffolds adds another level of complexity to AT_2_ receptor signaling. Furthermore, this heteromerization-mediated recruitment of β-arrestin could explain some of the contradictory studies that report AT_2_-mediated activation of MAPKs (65, 66).

Coexpression of the AT_2_ receptor and the B_2_ receptor in the same cell is of course a primary requisite for formation of a heteromer. Expression of both receptors in endothelial cells is well documented (37, 67), and as both receptors initiate endothelium-mediated vasodilation *via* the NO/cGMP pathway (59), this is a probable location that we may expect functional AT_2_-B_2_ heteromers to be present. This is further supported by the previous heteromer study, which found that heteromerization of these two receptors resulted in enhanced NO signaling (24). Both receptors are also found in smooth muscle cells of the vasculature and in the heart, indicating the potential for heteromer formation in these cells, and further allowing for a role for the heteromer in the cardiovascular system. Beyond the cardiovascular system, the two receptors are also coexpressed in uterine smooth muscle cells, epithelial cells and fibroblasts (59, 68, 69), suggesting a broad range of cells the heteromer may be present in.

Although the AT_2_ receptor does not interact with the traditional GPCR interacting proteins, it is, however, known to interact with other signaling and regulatory proteins at its intracellular face. Interactions with the ErbB3 epidermal growth factor receptor (70), the scaffold protein connector enhancer of Ksr (71) and tissue inhibitor of metalloproteinases-3 (72) are implicated in AT_2_ receptor-mediated antigrowth effects, while interactions with the transcription factor promyelocytic zinc finger protein are involved in the mediation of cardiac hypertrophy (73). Interactions with the Na^+^/H^+^ exchanger NHE6 are important for AT_2_ receptor regulation of sodium levels (74), and interactions with AT_2_ receptor-interacting protein 1 result in antigrowth effects (75, 76) and neural differentiation (77). It is possible that when the AT_2_ receptor is heteromerized with the B_2_ receptor, recruitment and interaction with the proteins investigated in this study (Gα_q_, Gα_z,_ GRK2 and β-arrestin2) could modulate the above AT_2_ receptor interactions, leading to alterations in signaling. In addition, many studies reveal that the AT_2_ receptor is constitutively active (65, 78–81). If recruitment and interaction of the proteins in this study to the AT_2_-B_2_ heteromer were able to block the interaction between the AT_2_ receptor and its various signaling partners, this would be a mechanism of reducing the constitutive activity observed for this receptor.

Despite decades of research, the AT_2_ receptor remains incompletely characterized in terms of its molecular pharmacology and its physiological functions (2, 3). Its lack of canonical GPCR pharmacology, such as agonist-induced G protein coupling, desensitization and internalization, make it a unique and enigmatic receptor within the field. The antagonist assays conducted in this study initially suggested that it has a somewhat silent role within the heteromer, as in every functional assay we investigated, B_2_ activation but not AT_2_ activation was required for the heteromer response. However, the Gα_z_ results demonstrate an important functional role of the AT_2_ receptor within the heteromer, as it confers novel Gα_z_ coupling to the B_2_ receptor. This is indicative of bi-directional modulation within the heteromer, as both the presence of the AT_2_ receptor and activation of the B_2_ receptor is required for modulation of Gα_z_ proximity.

In summary, we have provided evidence for the existence of the AT_2_-B_2_ heteromer and have demonstrated some of its apparent novel pharmacology. Extension of these findings beyond HEK293FT cells to more physiologically relevant systems will enable further characterization of the pharmacology mediated by the heteromer. Heteromerization of the AT_2_ and B_2_ receptors likely underpins some of the functional crosstalk observed between the receptors in the cardiovascular system, and it is possible that the heteromer may also have physiological roles in other areas of the body, such as the nervous system. AT_2_-B_2_ heteromerization is a newly identified mechanism for the enigmatic and poorly characterized AT_2_ receptor to be functionally active within cells.

## Data Availability Statement

The raw data supporting the conclusions of this article will be made available by the authors, without undue reservation.

## Author Contributions

EKMJ, MAA, RJH, HBS and RSA conducted the experiments and analyzed the results. EKMJ, MAA, RMS and KDGP conceived the experiments. EKMJ and KDGP wrote the paper. All authors reviewed the manuscript.

## Funding

This work was funded in part by the Australian Research Council (ARC; DP120101297 and FT100100271) and Dimerix Bioscience Pty Ltd. Dimerix was not involved in the study design, collection, analysis, interpretation of data, the writing of this article or the decision to submit it for publication. EJ was funded for part of this work by the Richard Walter Gibbon Medical Research Scholarship from The University of Western Australia and by an ARC Centre for Personalised Therapeutics Technologies (IC170100016) postdoctoral fellowship.

## Conflict of Interest

KP has a shareholding in Dimerix Limited, a spin-out company of The University of Western Australia that owns intellectual property relating to the Receptor-HIT technology and that partially funded this work. KP is Chief Scientific Advisor to Dimerix. KP, ES and RS are named inventors on patents covering the Receptor-HIT technology (WO/2008/055313 Detection System and Uses Therefor).

The remaining authors declare that the research was conducted in the absence of any commercial or financial relationships that could be construed as a potential conflict of interest.

## Publisher’s Note

All claims expressed in this article are solely those of the authors and do not necessarily represent those of their affiliated organizations, or those of the publisher, the editors and the reviewers. Any product that may be evaluated in this article, or claim that may be made by its manufacturer, is not guaranteed or endorsed by the publisher.
